# Impact of Individual, Organizational, and Technological Factors on the Implementation of an Online Portal to Support a Clinical Pathway Addressing Psycho-Oncology Care: Mixed Methods Study

**DOI:** 10.2196/26390

**Published:** 2021-04-14

**Authors:** Lindy Masya, Heather L Shepherd, Phyllis Butow, Liesbeth Geerligs, Karen C Allison, Colette Dolan, Gabrielle Prest, The ADAPT Program Group, Joanne Shaw

**Affiliations:** 1 Psycho-Oncology Co-operative Research Group School of Psychology The University of Sydney Sydney Australia; 2 Sydney Local Health District Camperdown Australia; 3 Centre for Medical Psychology and Evidence-based Decision-Making School of Psychology The University of Sydney Sydney Australia; 4 The Kinghorn Cancer Centre Sydney Australia; 5 Australian College of Nursing Sydney Australia; 6 College of Nursing and Health Sciences Flinders University Adelaide Australia; 7 see Acknowledgments

**Keywords:** decision support systems, clinical decision making, psycho-oncology, health informatics, clinical pathways, health services research

## Abstract

**Background:**

Clinical pathways (CPs) can improve patient outcomes but can be complex to implement. Technologies, such as clinical decision support (CDS) tools, can facilitate their use, but require end-user testing in clinical settings.

**Objective:**

This study applied the Technology Acceptance Model to evaluate the individual, organizational, and technological contexts impacting application of a portal to facilitate a CP for anxiety and depression (the ADAPT Portal) in a metropolitan cancer service. The ADAPT Portal triggers patient screening on patient reported outcomes, alerts staff to high scores, recommends evidence-based management, and triggers review and rescreening at set intervals.

**Methods:**

Quantitative and qualitative data on portal activity, data accuracy, and health service staff perspectives were collected. Quantitative data were analyzed descriptively, and thematic analysis was applied to qualitative data.

**Results:**

Overall, 15 (100% of those invited) health service staff agreed to be interviewed. During the pilot, 73 users (36 health service staff members and 37 patients) were registered on the ADAPT Portal. Of the 37 patients registered, 16 (43%) completed screening at least once, with seven screening positive and triaged appropriately. In total, 34 support requests were lodged, resulting in 17 portal enhancements (technical issues). Health service staff considered the ADAPT Portal easy to use and useful; however, some deemed it unnecessary or burdensome (individual issues), particularly in a busy cancer service (organizational issues).

**Conclusions:**

User testing of a CDS to facilitate screening and assessment of anxiety and depression in cancer patients highlighted some technological issues in implementing the ADAPT CDS, resulting in 17 enhancements. Our results highlight the importance of obtaining health service staff feedback when piloting specialized CDS tools and addressing contextual factors when implementing them.

## Introduction

In the last 25 years, health care has focused on improving the quality and value of care delivery through standardization of the management of specific conditions with guidelines and clinical pathways (CPs) [1,2]. CPs are structured, multidisciplinary, evidence-based management plans for a specific health condition. They outline the appropriate management with respect to clinical interventions, resources, timeframes, progress milestones, and expected outcomes, with the aim of standardizing improved co-ordination and continuity of patient care across different specialties and services [3].

The *Australian clinical pathway for the screening, assessment, and management of anxiety and depression in adults with cancer* (ADAPT CP) [4] highlights the need for routine psychological screening with appropriate follow-up for patients being treated for cancer. Cancer patients report a high unmet need for psychosocial care [5], and health professionals commonly underestimate or fail to detect patients’ psychosocial concerns [6]. Screening and follow-up of anxiety and depression improve patient adherence to cancer treatment, reduce health service utilization, improve quality of life, and reduce suffering, as well as decrease the risk of patients developing a major mood disorder [7-9]. The ADAPT CP provides a structured pathway for screening, assessing, and responding to anxiety and depression in cancer care to ensure optimal patient outcomes are achieved.

However, studies across numerous health conditions confirm that guidelines and CPs are not enough to change patient care within complex health systems owing to knowledge gaps, poor communication, and insufficient implementation efforts [2,10,11]. There is growing evidence that technology can facilitate the adherence of health care organizations to CPs. Clinical decision support (CDS) tools comprise computerized alerts, reminders, and standardized data collection formats to assist health professionals with clinical decision making at the point of care [12]. Earlier CDS tools were often cost prohibitive, utilized unvalidated tools, were disruptive to clinical care processes, provided inconsistent information, or were not presented at vital points in the clinical decision-making process [13]. However, more recent CDS tools have demonstrated the benefits of improved treatment management, reduced time to treatment, standardized data collection [14], reduced clinician documentation time, lower medication errors, reduced adverse drug events [15], and greater guideline adherence [16-18]. Our group recently developed a CDS for ADAPT (the ADAPT Portal) to optimize ease of delivery of the ADAPT CP and ensure all patients receive care according to the CP.

Several theoretical models have been proposed to explain uptake and guide assessment of CDS tools, and the most widely used is the Technology Acceptance Model [19] for assessing health care technology uptake [20,21]. This model (an adaptation of Fishbein and Ajzen’s theory of reasoned action [22]) presumes a mediating role of perceived ease of use and usefulness in association with system characteristics (external variables) for explaining system uptake and usage. Perceived usefulness is defined as the degree to which a user believes that using a specific system will enhance the job performance, while perceived ease of use is defined as the degree to which a user believes that using a particular system will be effort free. External variables have been less well defined, but include aspects, such as user experience and role, and external factors in the work environment that impact usage.

This study sought to apply the Technology Acceptance Model in a pilot of the ADAPT Portal with target end users to rigorously evaluate its utility prior to a large-scale evaluation of the ADAPT CP overall. Our aim was to refine the system to best meet users’ needs prior to a large-scale implementation of the ADAPT Portal. More specifically, the study aimed to evaluate the individual, organizational, and technological contexts impacting the ADAPT Portal’s perceived usability, usefulness, and appropriateness within a clinical cancer service.

## Methods

### Study Setting and Design

The study was conducted in a cancer service within a large Australian metropolitan hospital. The cancer service elected to include patients receiving chemotherapy as part of their care in the study.

A triangulation mixed methods design [23] was employed. It combined qualitative and quantitative data sources to obtain different but complementary data to best understand these issues.

### Recruitment Procedure

After senior management confirmed participation in the study and a research participation agreement was established with the cancer service, a subset of health service staff at the oncology service (purposively selected to ensure diversity in professional backgrounds and ADAPT CP roles) was invited to participate in the study. Staff received an email from the study team inviting them to participate and provide written informed consent. Participating staff were interviewed after the implementation period to capture their experience of planning for and using the ADAPT Portal within their service.

All patients commencing treatment during the study period at the site were invited to participate in the study. Interested patients provided written consent to participate in ADAPT screening and allow the research team to access their medical records.

### Study Procedure

A lead team comprising management staff, nursing staff, social work staff, psychology staff, clinical system specialists, medical oncology specialists, and service improvement staff worked with the research team to tailor the ADAPT Portal to their local needs, resources, and preferences. The lead team mapped the CP and cancer service operations and compiled these into a workflow that operationalized how the ADAPT Portal would be used at the center. User training on the tailored ADAPT CP and Portal was provided to medical oncology, nursing, and allied health staff, with key ADAPT Portal users attending individual training sessions according to their roles and responsibilities in the ADAPT CP and associated tasks within the ADAPT Portal.

The ADAPT CP was then implemented for 5 months among several tumor streams within the medical oncology service. During implementation, users (health service staff and patients) had access to online, phone, and email support from the research team. After implementation, staff interviews were carried out, and portal usage and contacts with the research team were collated.

The study was approved by the Human Research Ethics Committee of the participating health care institution.

### ADAPT Portal

The ADAPT Portal was developed by a multidisciplinary working group (comprising psycho-oncologists, oncologists, researchers, patient representatives, and information technology [IT] web designers and programmers) tasked with defining the ADAPT Portal’s scope and functionality via agile design [24]. The goal was to operationalize the ADAPT CP [4] to make it as easy as possible for cancer services to enact within current workflows. A task analysis was conducted to identify required user interactions and data elements. This allowed tasks (dialogue between users and the system) to be grouped into modules that framed the functionality of the system (registration, screening, triage, referral, progress review, and rescreening). Components of the system that could be automated to reduce workload and facilitate health professional action where required (eg, via notifications, alerts, reminders, and reports) were identified. Complex algorithms were developed to cover all contingencies to ensure the CP was appropriately enacted for all patients. Visual mock-ups were iteratively developed and reviewed for flow and an optimal interface. User access levels were set to ensure privacy and confidentiality.

The web-based ADAPT Portal ultimately consisted of two parts. The first part was a patient-directed portal where patients verify their registration and create a password to activate their portal account, and are directed to the home page where information and resources are available. At scheduled time points, patients receive an email alert with a direct link to complete anxiety and depression screening measures and can access self-management and information resources. The second part was a health service staff portal where health service staff log in using a password, register patients who have agreed to participate in the CP with their contact details, receive alerts of patients scoring above clinical cutoffs, and are prompted to complete evidence-based actions according to CP recommendations. Clinical staff can visually track patients’ longitudinal screening data and CP progression, as well as generate reports at an individual or service level. Links to education and training resources are accessible to staff via the portal along with portal user guides and a support messaging service.

### Measures

#### Quantitative Data Collection

ADAPT Portal user activity was reviewed to identify system functionality and uptake. A random selection of registered consenting patients’ medical records was reviewed to assess the quality of data captured and discrepancies between CP documentation in the ADAPT Portal and patients’ electronic medical records.

During the 5-month implementation, user support contacts were tracked, capturing the reason for contact and duration of support required. This information was reviewed and coded according to the ADAPT Portal functional domains (ie, registration, screening, referral, review, rescreening, user error, and system error) for analysis. Additionally, potential design improvements identified during lead team meetings, training sessions, and user support contacts to improve system performance and user satisfaction of the ADAPT Portal were logged throughout the study. These were reviewed by the study team and classified as *critical* (potential cause of system breakdown), *serious* (cause of frustration and nonengagement, but not critical to system function), or *minor* (mostly cosmetic issues that were not of major concern to staff).

#### Qualitative Data

Data were obtained via health service staff user interviews, review of user support contacts, and field observations by the ADAPT research team. Using purposive sampling, 15 health service staff members participated in semistructured interviews with an interviewer independent of the core ADAPT research team. Interviews explored perceived acceptability and utility of the ADAPT Portal, problems and challenges encountered with the system, and recommendations for improvement. Interviews were transcribed for analysis. Additional data from the staff interviews focusing on staff and organizational barriers to utilizing the ADAPT CP are published elsewhere [25]. The ADAPT research team also recorded extensive field observations after each user support contact with staff as well as during meetings with the lead team during the implementation process to record issues raised and resolutions reached.

### Analysis

Quantitative data were entered into the Statistical Package for the Social Sciences (SPSS) database. Descriptive statistics (means and medians for continuous data and percentages for categorical data) were generated.

Interview transcripts were thematically analyzed by two researchers using the platform NVIVO. The two researchers independently performed initial coding to group information according to the modified Technology Acceptance Model themes [19-21] as follows: (1) individual context, individual user’s perceptions about compatibility and attitude toward the ADAPT Portal; (2) organizational context, facilitators of acceptance such as infrastructure, support, and social norms; and (3) technological context, perceived ease of use, problems reported, and change in habits resulting from using the ADAPT Portal. Any disagreements were resolved through discussion and consensus. Thematic analysis was then applied within each category to further refine the themes [26]. Each coder read six transcripts and generated a draft coding tree to capture the underlying meaning of the text, which was discussed until consensus was reached. The coding tree was iteratively revised after further coding. The text was compared and contrasted with existing themes until a final comprehensive coding structure was achieved, and the remaining transcripts were then coded.

## Results

### Portal Users

A total of 73 ADAPT Portal users (36 health service staff and 37 patients) were registered on the ADAPT Portal during the pilot, of whom 67 (92%) accessed the Portal.

### Health Service Staff Participants

Registered health service staff included one administrator, two data managers, eight medical oncologists, 13 registered nurses, three cancer care coordinators, one clinical nurse specialist, one clinical nurse educator, four clinical psychologists, and three social workers. Of these, 15 were purposively selected (to ensure diversity of background and ADAPT CP roles) to participate in the postimplementation interview (all agreed). The interview sample included both full-time and part-time staff, who had been in their current role for an average of 3 years (Table 1).

**Table 1:** Interviewee demographic profile.

Demographic		Total (n=15)
**Age, n (%)**		
	26-50 years	12 (80%)
	51-75 years	3 (20%)
**Gender, n**		
	Female	15
**Role, n**		
	Oncologist	1
	Nurse-RN^a^	2
	Nurse-CNS^b^, CNC^c^, coordinator	3
	NUM^d^/clinical managers	3
	Clinical psychologist	3
	Social worker	1
	Clinical trial manager	1
	Data manager	1
Duration in the current role, mean (range)		3.4 years (5 months to 10 years)
**Employment status, n (%)**		
	Full time	9 (60%)
	Part time	6 (40%)

^a^RN: registered nurse.

^b^CNS: clinical nurse specialist.

^c^CNC: clinical nurse consultant.

^d^NUM: nursing unit manager.

### Portal Usage

Of the 37 patients registered, 16 (43%) completed screening once, with seven screening positive. In response to system alerts sent to nominated clinical staff, staff triaged all seven patients. Following triage, the step allocation for two patients was downgraded and documented in the ADAPT Portal, two patients declined additional support, and three patients were referred via the ADAPT Portal to psychosocial services (Table 2).

**Table 2:** Portal user activity.

Portal activity	Patients, n	Health service staff, n
Number registered	37	36
Number accessed the portal	35	32
Number screened	16	N/A^a^
Total number of screening events	17	N/A
Total number of positive screens	7	N/A
Number of patients triaged	7	N/A
Number of referrals	3	N/A

^a^N/A: not applicable.

### Support Requests and Suggested IT Improvements

A total of 34 research support requests were lodged during the 5-month implementation period, with the majority lodged by health service staff (n=32, 94%) and 2 (6%) by patients. Table 3 lists the types of support requests lodged. Over a third requested clarification regarding management of patient scenarios in alignment with the CP/Portal workflow (n=13, 38%), including screening (n=5), registering (n=4), triage (n=3), and referral (n=1). The remaining support contacts lodged by health service staff were related to user errors, such as requesting password resets (n=7, 21%), system or network errors, such as Wi-Fi dropout (n=6,18%), health service set-up and configuration issues, such as health service staff not verifying accounts (n=3, 9%), and staff training (n=3, 9%). Usability was raised in two support requests around user habits of pressing “Enter” to move between fields, which in the ADAPT Portal, triggered field validation prompts and cleared input data from some fields.

**Table 3:** Summary of unplanned support contact.

Support contact domain	Total (n=34), n
Workflow	13
User error	7
System & network error	6
Set-up & configuration	3
Training	3
Usability	2

Regular review of support contacts and researcher observations led to 17 suggestions for improvements in the system, and of these, five were classified as critical and four were classified as serious (Table 4). Most identified improvements pertained to screening (n=5), reporting (n=4), and patient registration (n=4) functionality. However, other improvements were identified in the triage (n=2), system configuration (n=1), and referral (n=1) functional domains. Examples included additional reporting items to record the reasons why patients did not complete screening, the ability to resend user registration emails to staff who had not verified their accounts, and allowing the “Start Screening” button to continuously display until the patient completed screening (to account for rescheduled appointments and other delays).

**Table 4:** Summary of system improvements.

Functionality domain	Severity, n			
	Critical	Serious	Minor	Total (n=17)
Reporting	N/A^a^	1	3	4
Screening	2	2	1	5
Patient registration	N/A	1	4	4
Triage functionality	1	N/A	1	2
Configuration	1	N/A	N/A	1
Referral	1	N/A	N/A	1

^a^N/A: not applicable.

### Portal Data Accuracy

Ten patients’ electronic medical records (EMRs) were compared with ADAPT Portal extracts to evaluate data capture and accuracy. These highlighted frequent missing or incorrect data on cancer diagnosis date and cancer staging in the ADAPT Portal, which occurred when these data were not available in the EMR system at the time of patient registration and were not subsequently updated in the ADAPT Portal when the information became available. CP activity recorded in the ADAPT Portal was consistent with actual psychosocial care documented in the EMR, except in two cases where the patients refused treatment. In these cases, users did not document this via the ADAPT Portal referral functionality, but rather as a free text note similar to current EMR documentation practice.

### Individual Views on Usability

Interview length ranged from 16 to 50 minutes (average, 25 minutes), and the themes identified focused on usability and views of ADAPT Portal processes. Staff reported that the system was easy to use and navigate as follows:

I’m not very tech savvy, but it was fine, it was very easy.

Interview participant #5 (i5), nursing unit manager/clinical manager

However, some staff reported difficulty logging into the ADAPT Portal owing to forgetting their passwords or poor Wi-Fi connectivity, while others reported that the time lag between training and actually using the system was too long, impacting their ADAPT Portal use confidence. Nevertheless, these challenges were quickly overcome as shown in the following comment:

By the time we got a referral we thought, oh how do we do this? How do we log in? What do we do? But, it was fine – you know, we figured it out and we could email [the support team] and she helped us.

i3, social worker

Staff also commented positively on system support, preferring this to user guides. One staff user made the following comment:

Contact was good – if staff asked team for resources or help, response was prompt.

i15, psychologist

Feedback on the usefulness of the ADAPT Portal for patient care was polarized. Some staff believed the ADAPT Portal did not improve on existing service processes that were well established, demonstrated in this comment:

So I think it [the ADAPT Portal] has a very good role but we’re already covering those areas.

i9, nurse-clinical nurse specialist, clinical nurse consultant, coordinator

Others reported that the ADAPT Portal was a useful mechanism to formally document psychosocial processes and remind staff that psychosocial assistance was part of standard patient care. One participant clarified their view:

I think we need to probably formalize what processes we've already got in place…I think it's important we're doing it with all patients, it’s part of the ongoing assessment of them.

i11, nursing unit manager/clinical manager

Staff endorsed the patient resources containing local and national support information, as patients could access relevant information in one location at their own convenience. An example of a comment made by participants was:

It's useful to have and it's good for the patient.

i3, social worker

Staff reported varied responses from patients, with most patients open to and positive about using the ADAPT Portal, but others rejected routine screening as unnecessary or too complex. One staff user observed an elderly patient having trouble screening via a tablet and decided to abandon screening.

### Organizational Context

Staff reported the need for the ADAPT Portal to be linked with the existing EMR as staff already log into multiple systems for patient care and other patient screening assessments are integrated into the EMR. Participants noted that the service has undergone major technological change in the last 2 years and were therefore reluctant to undertake further technological change. This was highlighted in the following comment:

We’ve only had that I think, just two years or, so we’ve just had a massive change with that, when everybody made electronic referrals and things, and I guess maybe this was just another thing that was put onto people

i10, nurse-clinical nurse specialist, clinical nurse consultant, coordinator

### Technological Context

Staff reported that their work habits changed during the implementation period because they had to access an additional system, and their workload increased. For one user, the role expanded. Regarding the ADAPT Portal, a health service staff member made the following comment:

...was an extra thing that you’re being asked to do.

i1, nurse-registered nurse

The service found it necessary to nominate one nurse to remind staff when their patients were due for screening, despite the ADAPT Portal automatically alerting staff, to ensure screening was completed, as summarized in the comment below:

Even though there’s a reminder we still forget sometimes. So, I think that one person [overseeing] is good.

i2, nurse-registered nurse

## Discussion

### Principal Findings

This study is the first to review an online clinical decision support system for a CP addressing anxiety and depression screening and management (the ADAPT Portal) in an Australian cancer service. We assessed the individual, organizational, and technological contexts impacting the ADAPT Portal’s perceived usability, usefulness, and appropriateness, and adjusted the system where possible to facilitate uptake in a larger implementation study. This is a critical step in the development of new systems for use in clinical care, and is rarely evaluated qualitatively and quantitatively.

Testing the system, responding to staff support contacts, making changes to the CDS, and providing training in altered processes and components took some time and delayed patient registrations for some weeks. Ultimately, 37 patients were successfully registered, and their progress through the system was tracked.

Our study highlighted a number of usability issues, technical barriers, and training requirements that resulted in 17 improvements to the ADAPT Portal. Improvements to the ADAPT Portal allowed better recording of the rationale behind decisions and adjustment for real-world variations in patient flow through the system. These findings highlight the importance of addressing perceived usability to ensure the smooth delivery of CDS tools, such as the ADAPT CP, and mirror findings from other studies on diverse CDS tools (such as a movie recommendation system [27], social networking system [28], and health care information system [29]) that have found usability to be a key factor in determining uptake.

Nevertheless, while a number of usability issues were revealed and rectified during the study, staff on the whole had positive perceptions regarding the usefulness of the ADAPT Portal to their patients and the oncology service, which proved to be a strong motivator for ongoing use of the portal. This finding further supports the validity of the Technology Acceptance Model and reflects findings from previous studies [20,21], which have reinforced the importance of perceived usefulness in determining the uptake of health-related technology. As ease of use has been shown to impact perceived usefulness [30], both variables are clearly key to ensuring the successful introduction of technology into diverse workplaces, including the health system.

Not all staff perceived the ADAPT CP to be useful in their practice. Some believed that their existing internal processes were already effective in identifying patients requiring psychosocial support, thus rendering the ADAPT Portal unnecessary in their eyes. In contrast, 7 of 16 patients screened on the ADAPT Portal scored in the range requiring triage and referral, and may have been missed without the system in place. The PARiHS implementation framework, commonly applied to health service change efforts, suggests that staff require evidence of intervention efficacy from not only randomized controlled trials, but also their own and patient experiences, and local evidence of needs and benefits [31]. Thus, finding clear ways to communicate local benefits to staff is vital to implementation success.

While ADAPT Portal usability was addressed in this study and staff were positive about the system on the whole, some contextual issues remained as barriers. These included our inability to integrate the portal into the established electronic record management system, which increased staff burden in learning and accessing an additional system. Furthermore, staff had only recently experienced a sharp learning curve in adapting to a new EMR, reducing their capacity to learn another. James Tcheng from the US National Academy of Medicine [13] noted that technology is primarily useful for “its potential to ameliorate the burden that exponentially expanding clinical knowledge as well as care and choice complexity place on the finite time and attention of clinicians, patients, and every other member of the care team.” Thus, it remains important to ensure that technology realizes this promise by ultimately reducing burden. Furthermore, this finding reinforces the utility of measuring external factors, as well as perceived usability and usefulness in assessing technology implementation.

This study had a number of strengths, including a mixed methods design that produced a rich and complementary data set and the use of a recognized model for evaluating technology acceptance. A number of study limitations must also be considered. This was a small pilot in one urban site and may not reflect findings in other oncology services, including those in small rural areas. Implementation was for 5 months, and some issues related to technology usability may not have arisen in that time. Evaluation over a longer implementation period is required.

### Conclusion

As a clinical decision support system, the ADAPT Portal achieved its goal in aligning patient care at a metropolitan cancer service with the recommendations of the ADAPT CP [4]. The pilot study results revealed that staff perceived the ADAPT Portal to be easy to use, and identified system improvements around design and additional functionality to increase usability, performance, and user satisfaction of the system at point of care. The usefulness of the ADAPT Portal was acknowledged by staff; however, some deemed it unnecessary or too burdensome, highlighting the importance of contextual factors when implementing change. The findings were invaluable for the research team in terms of refining the ADAPT Portal and structuring the implementation strategies and other supporting resources planned for evaluation in a large-scale implementation trial with cancer services [32]. Results of the large-scale implementation study will provide evidence of the effectiveness of the ADAPT Portal as a CDS system for bringing about large-scale adherence to evidence-based practice within cancer services and in differing contexts.

## Abbreviations

ADAPT CP: Australian clinical pathway for the screening, assessment, and management of anxiety and depression in adults with cancer
CDS: clinical decision support
CP: clinical pathway
EMR: electronic medical record
IT: information technology

## References

[1] Middleton B, Sittig DF, Wright A (2016). Clinical Decision Support: a 25 Year Retrospective and a 25 Year Vision. Yearb Med Inform.

[2] Gross PA, Greenfield S, Cretin S, Ferguson J, Grimshaw J, Grol R, Klazinga N, Lorenz W, Meyer GS, Riccobono C, Schoenbaum SC, Schyve P, Shaw C (2001). Optimal Methods for Guideline Implementation. Medical Care.

[3] Kinsman L, Rotter T, James E, Snow P, Willis J (2010). What is a clinical pathway? Development of a definition to inform the debate. BMC Med.

[4] Butow P, Price MA, Shaw JM, Turner J, Clayton JM, Grimison P, Rankin N, Kirsten L (2015). Clinical pathway for the screening, assessment and management of anxiety and depression in adult cancer patients: Australian guidelines. Psychooncology.

[5] Sanson-Fisher R, Girgis A, Boyes A, Bonevski B, Burton L, Cook P (2000). The unmet supportive care needs of patients with cancer. Cancer.

[6] Fallowfield L, Ratcliffe D, Jenkins V, Saul J (2001). Psychiatric morbidity and its recognition by doctors in patients with cancer. Br J Cancer.

[7] Howell D, Olsen K (2011). Distress-the 6th vital sign. Curr Oncol.

[8] Andersen BL, DeRubeis RJ, Berman BS, Gruman J, Champion VL, Massie MJ, Holland JC, Partridge AH, Bak K, Somerfield MR, Rowland JH, American Society of Clinical Oncology (2014). Screening, assessment, and care of anxiety and depressive symptoms in adults with cancer: an American Society of Clinical Oncology guideline adaptation. J Clin Oncol.

[9] Howell D, Keshavarz K, Broadfield L, Hack T, Hamel M, Harth T, Jones J, McLeod D, Olson K, Phan S, Sawka A, Swinton N, Ali M (2015). A pan Canadian practice guideline for screening, assessment, and management of cancer-related fatigue in adults (Version 2). Canadian Partnership Against Cancer (Cancer Journey Advisory Group) and the Canadian Association of Psychosocial Oncology.

[10] Shiffman RN, Michel G, Essaihi A, Thornquist E (2004). Bridging the guideline implementation gap: a systematic, document-centered approach to guideline implementation. J Am Med Inform Assoc.

[11] Zikmund-Fisher BJ, Kullgren JT, Fagerlin A, Klamerus ML, Bernstein SJ, Kerr EA (2017). Perceived Barriers to Implementing Individual Choosing Wisely Recommendations in Two National Surveys of Primary Care Providers. J Gen Intern Med.

[12] Heekin AM, Kontor J, Sax HC, Keller MS, Wellington A, Weingarten S (2018). Choosing Wisely clinical decision support adherence and associated inpatient outcomes. Am J Manag Care.

[13] Tcheng J, Bakken S, Bates DW, Bonner III H, Gandhi TK, Josephs H, Kawamoto K, Lomotan EA, Mackay E, Middleton B, Teich JM, Weingarten S, Hamilton Lopez M (2017). Optimizing Strategies for Clinical Decision Support: Summary of a Meeting Series.

[14] Gibbs J, Sutcliffe LJ, Gkatzidou V, Hone K, Ashcroft RE, Harding-Esch EM, Lowndes CM, Sadiq ST, Sonnenberg P, Estcourt CS (2016). The eClinical Care Pathway Framework: a novel structure for creation of online complex clinical care pathways and its application in the management of sexually transmitted infections. BMC Med Inform Decis Mak.

[15] Lenz R, Blaser R, Beyer M, Heger O, Biber C, Bäumlein M, Schnabel M (2007). IT support for clinical pathways--lessons learned. Int J Med Inform.

[16] Campanella P, Lovato E, Marone C, Fallacara L, Mancuso A, Ricciardi W, Specchia ML (2016). The impact of electronic health records on healthcare quality: a systematic review and meta-analysis. Eur J Public Health.

[17] Kawamoto K, Houlihan CA, Balas EA, Lobach DF (2005). Improving clinical practice using clinical decision support systems: a systematic review of trials to identify features critical to success. BMJ.

[18] Schedlbauer A, Prasad V, Mulvaney C, Phansalkar S, Stanton W, Bates DW, Avery AJ (2009). What evidence supports the use of computerized alerts and prompts to improve clinicians' prescribing behavior?. J Am Med Inform Assoc.

[19] Davis FD (1989). Perceived Usefulness, Perceived Ease of Use, and User Acceptance of Information Technology. MIS Quarterly.

[20] Orruño E, Gagnon MP, Asua J, Ben Abdeljelil A (2011). Evaluation of teledermatology adoption by health-care professionals using a modified Technology Acceptance Model. J Telemed Telecare.

[21] Buenestado D, Elorz J, Pérez-Yarza EG, Iruetaguena A, Segundo U, Barrena R, Pikatza JM (2013). Evaluating acceptance and user experience of a guideline-based clinical decision support system execution platform. J Med Syst.

[22] Fishbein M, Ajzen I (1975). Belief, attitude, intention and behavior: An introduction to theory and research.

[23] Creswell JW, Plano Clark VL, Gutmann M, Hanson W, Tashakkori A, Teddlie C, Teddlie CB (2003). Advanced mixed methods research designs. Handbook of Mixed Methods in Social & Behavioral Research.

[24] Moran A (2014). Agile Risk Management.

[25] Geerligs L, Shepherd H, Rankin N, Masya L, Shaw JM, Price MA, Dhillon H, Dolan C, Prest G, Butow P, ADAPT Program Group (2020). The value of real-world testing: a qualitative feasibility study to explore staff and organisational barriers and strategies to support implementation of a clinical pathway for the management of anxiety and depression in adult cancer patients. Pilot Feasibility Stud.

[26] Braun V, Clarke V (2006). Using thematic analysis in psychology. Qualitative Research in Psychology.

[27] Armentano MG, Christensen I, Schiaffino S (2015). Applying the Technology Acceptance Model to Evaluation of Recommender Systems. Polibits.

[28] Choi G, Chung H (2013). Applying the Technology Acceptance Model to Social Networking Sites (SNS): Impact of Subjective Norm and Social Capital on the Acceptance of SNS. International Journal of Human-Computer Interaction.

[29] Pai F, Huang K (2011). Applying the Technology Acceptance Model to the introduction of healthcare information systems. Technological Forecasting and Social Change.

[30] Shroff RH, Deneen CC, Ng EMW (2011). Analysis of the technology acceptance model in examining students' behavioural intention to use an e-portfolio system. AJET.

[31] Rycroft-Malone J, Seers K, Titchen A, Harvey G, Kitson A, McCormack B (2004). What counts as evidence in evidence-based practice?. J Adv Nurs.

[32] Butow P, Shaw J, Shepherd HL, Price M, Masya L, Kelly B, Rankin NM, Girgis A, Hack TF, Beale P, Viney R, Dhillon HM, Coll J, Kelly P, Lovell M, Grimison P, Shaw T, Luckett T, Cuddy J, White F, ADAPT Program Group (2018). Comparison of implementation strategies to influence adherence to the clinical pathway for screening, assessment and management of anxiety and depression in adult cancer patients (ADAPT CP): study protocol of a cluster randomised controlled trial. BMC Cancer.

